# Supplementary material for: Interplay between hydrophilicity and surface barriers on water transport in zeolite membranes
Source: Nat Commun. 2016 Oct 3;7:12762. doi: 10.1038/ncomms12762 (PMC5477497; doi:10.1038/ncomms12762)
Supplement: Supplementary Information — Supplementary Figures 1-11, Supplementary Notes 1-6, Supplementary Methods and Supplementary References [file ncomms12762-s1.pdf]

SUPPLEMENTARY FIGURES

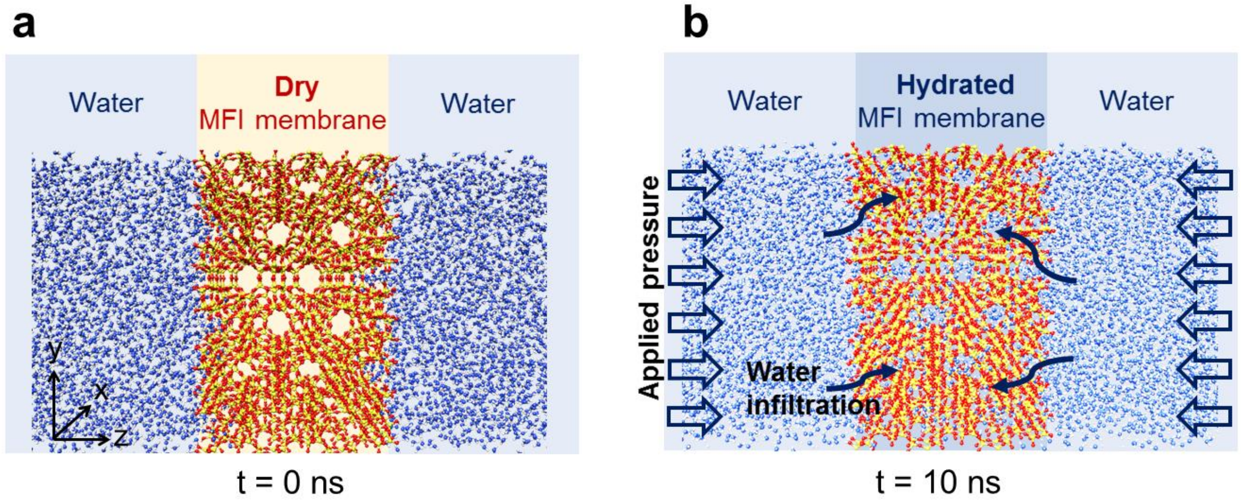

Supplementary Figure 1. Molecular dynamics setup for infiltration simulations. Schematic of the MD simulation box: silicalite-1 membrane (red/yellow) and water molecules (blue) are represented. **(a)** Detail of the silicalite-1 membrane before ($t = 0$ ns) and **(b)** after the water infiltration ($t = 10$ ns in the considered case) induced by water pressurization. Rendering pictures are made with UCSF Chimera. [1]

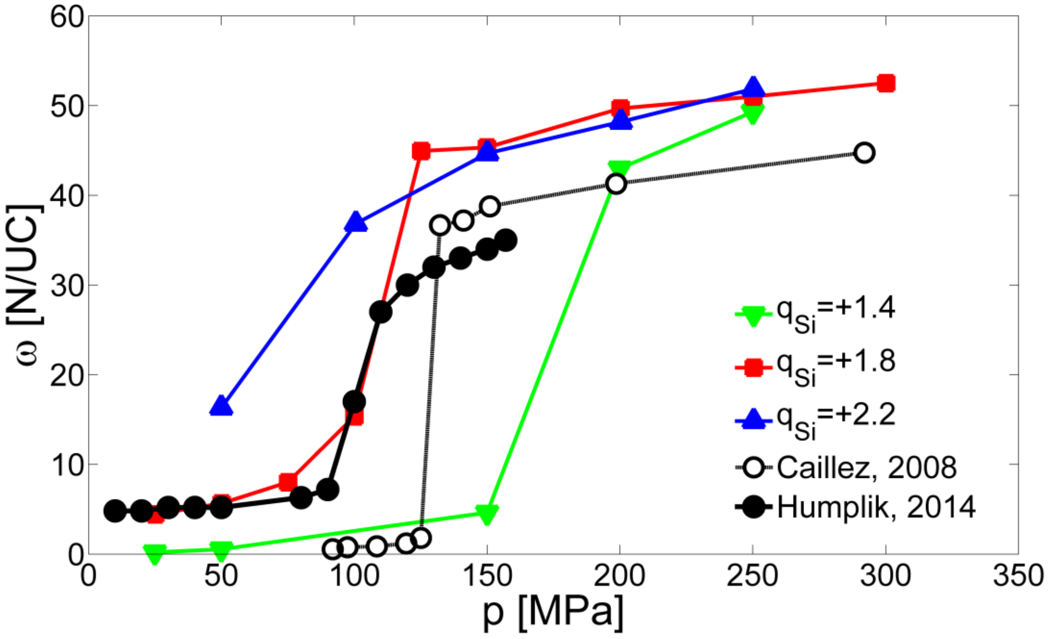

Supplementary Figure 2. Infiltration isotherms ($T = 300$ K) of water in a silicalite-1 membrane. Results from current MD simulations with different silicon partial charge ($q_{\mathrm{Si}} = 1.4$ e, green triangles; 1.8 e, red squares; 2.2 e, blue triangles) are compared with either Monte Carlo simulations from Cailliez *et al.* (open circles) or experiments by Humplik *et al.* (black circles).[2,3] Note that the maximum framework capacity (ω_{M}) of the structure, which is the plateau amount of infiltrated water molecules achieved at the maximum steric occupation of the accessible pore volume, is larger than the experimental evidence reported by Humplik *et al.*, namely 52 N/UC vs. 35 N/UC, respectively. [4] However, similar values have been found in other experimental (*i.e.*, 53 N/UC [5]) and numerical studies (*i.e.*, 57 N/UC [6]). The latter discrepancies with experiments may be due to the experimental analysis of imperfect zeolite specimens, which can present surface barriers, pore blockage or contamination of the structure and thus modifications of the accessible pore volume. However, changes in ω_{M} do not affect the dependence between infiltration pressures (*i.e.*, infiltration type), adsorption capacity and structure hydrophilicity, which is the focus of this analysis. [3,7]

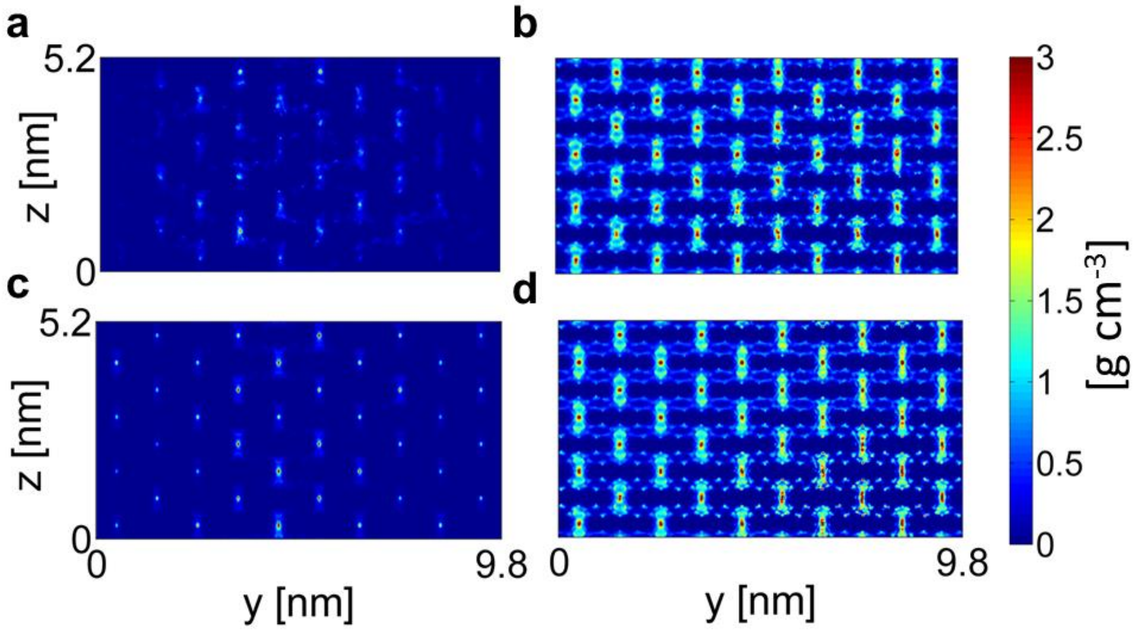

Supplementary Figure 3. Radial 2D density maps of water in MFI crystals. Values are averaged along x axis. **(a)** 0% Al/Si and $\vartheta_{\mathrm{M}} = 0.10$. **(b)** 0% Al/Si and $\vartheta_{\mathrm{M}} = 0.95$. **(c)** 3.06% Al/Si and $\vartheta_{\mathrm{M}} = 0.10$. **(d)** 3.06% Al/Si and $\vartheta_{\mathrm{M}} = 0.95$.

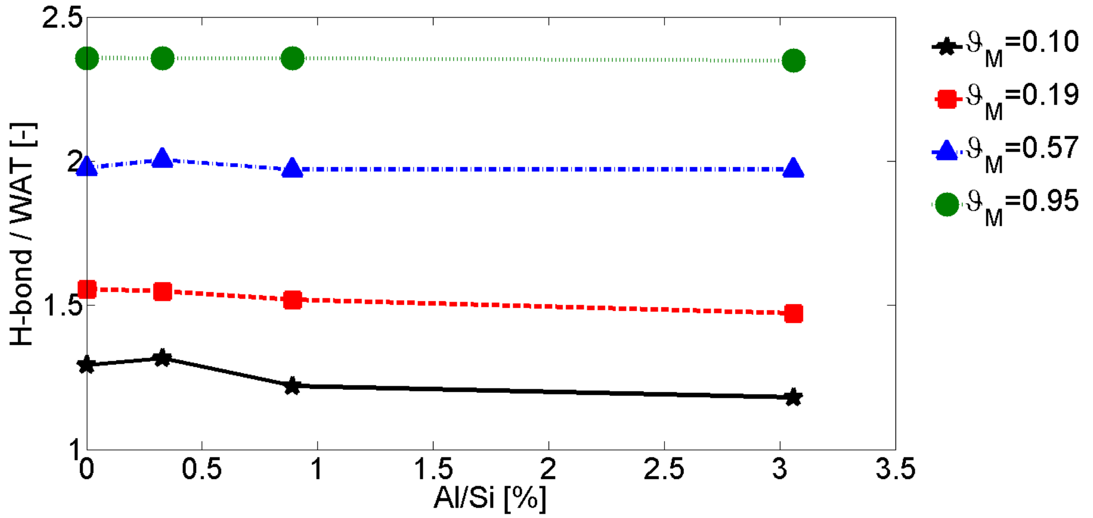

Supplementary Figure 4. Hydrogen bonds. Average amount of hydrogen bonds per water molecule with different pore hydration (ϑ_M) and increasing defect density (%Al/Si) in the considered defected MFI crystals with "weak" silanol nests. A general decrease in the average number of water-water H-bonds due to nanoconfinement (from typical values of 5-6 in bulk water to 1.3-2.4 with the confined zeolite structure) is observed. [3] The results demonstrate that both the *D* reduction and *D* insensitivity with respect to pore hydrophilicity at large ϑ_M mainly originate from water-water interactions. On the contrary, the *D* reduction with pore hydrophilicity at low ϑ_M can be attributed to the solid-water interactions.

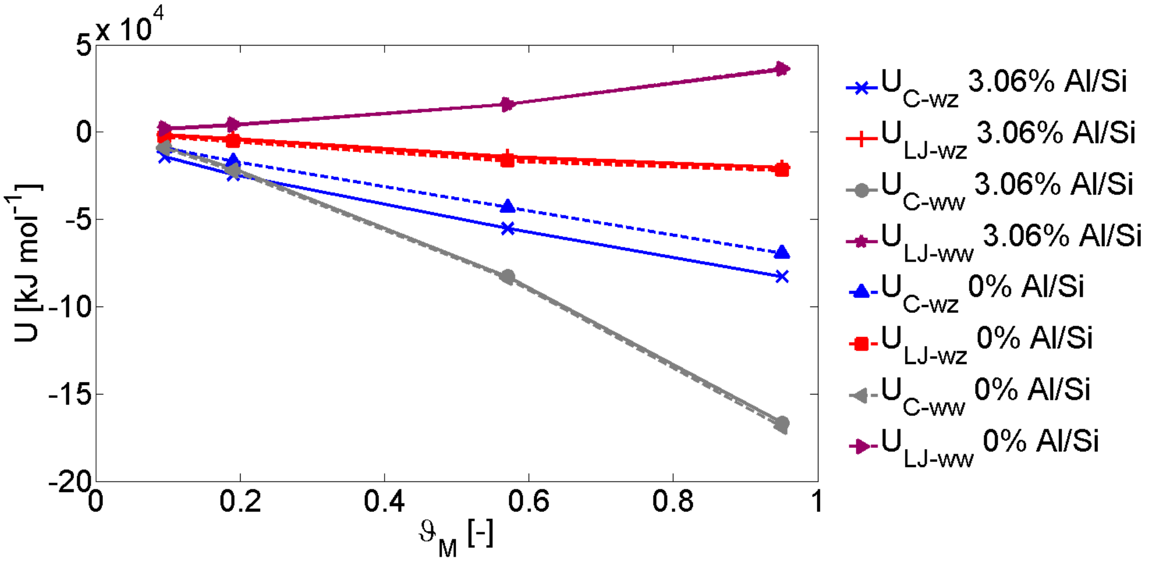

Supplementary Figure 5. Water-zeolite and water-water interactions. Water-zeolite (wz) and water-water (ww) non-bonded interaction energies for MFI crystals with 0% (dashed lines) and 3.06% (solid lines) Al/Si defect concentrations, at increasing pore hydration. Both Coulomb (U_{C}) and Lennard-Jones (U_{LJ}) contributes to the effective interaction potential ($U_{\mathrm{eff}} = U_{\mathrm{C}} + U_{\mathrm{LJ}}$) are depicted. Note that the absolute value of all of the energy contributions increases with higher ϑ_{M}, because of the larger amount of water molecules infiltrated in the zeolite pores. The increased hydrophilicity of the MFI crystal can be seen by the enhancement of $U_{\mathrm{C-wz}}$ (which shows larger negative values in the case of a more defective zeolites). This absolute increase in $U_{\mathrm{C-wz}}$ for the more hydrophilic pores is constant at different ϑ_{M} values. Therefore, at low pore hydrations, the $U_{\mathrm{C-wz}}$ enhancement has a significant contribution when compared with the other energy components (it can also be seen that it becomes negligible at large ϑ_{M} because of the strong increase in water-water interactions such as $U_{\mathrm{LJ-ww}}$ and $U_{\mathrm{C-ww}}$).

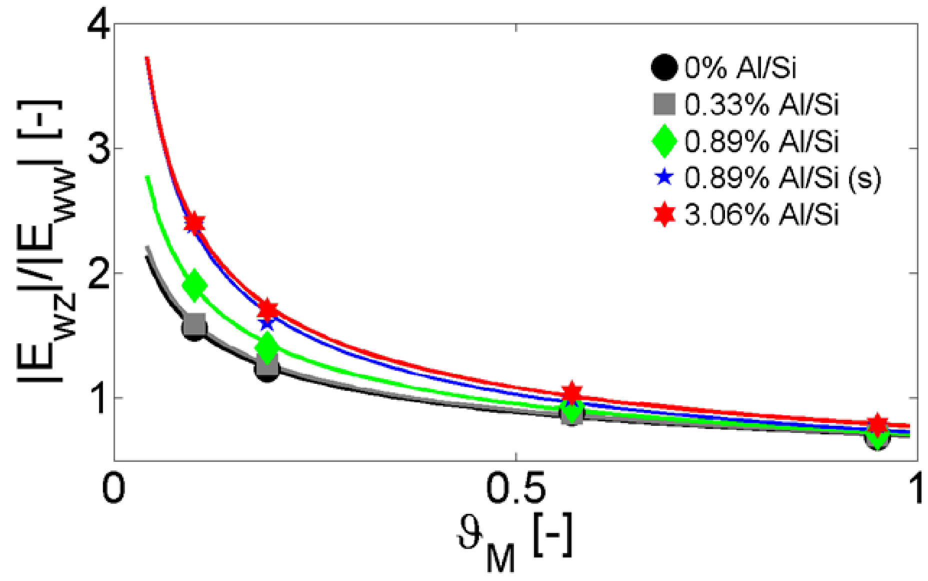

Supplementary Figure 6. Interaction energies ratio. Ratio between the absolute values of water-zeolite E_{wz} and water-water E_{ww} specific interaction energies (see Supplementary Equations (1) and (2)). MFI crystals with different Al/Si defect concentration and pore hydration are considered. MD results are fit by decaying power laws (solid lines).

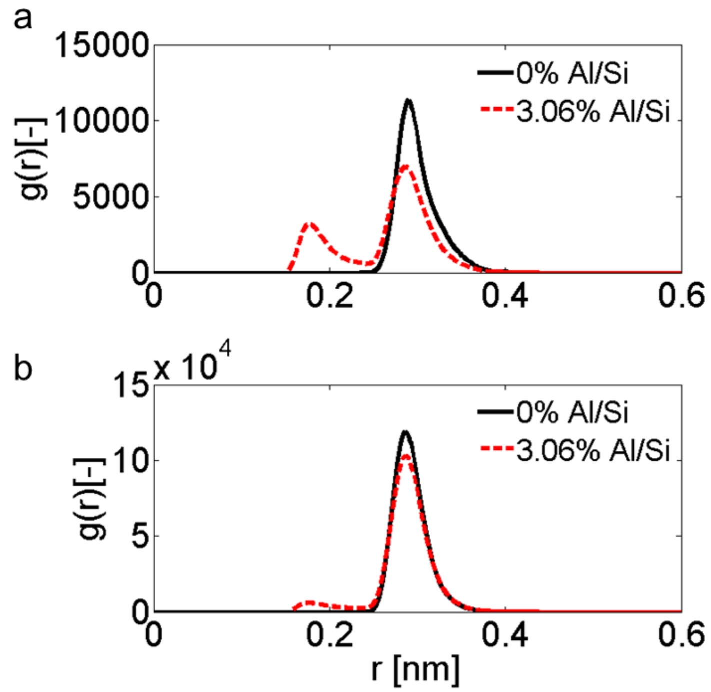

Supplementary Figure 7. Radial distribution functions of water. Radial distribution functions of water (oxygen atoms are considered as representative for the whole water molecule) with respect to the inner surface of the pores: a comparison between pristine and defected (3.06% Al/Si) MFI crystals is shown. **(a)** $\vartheta_{\mathrm{M}} = 0.10$; **(b)** $\vartheta_{\mathrm{M}} = 0.95$. Note that for the more hydrophilic membrane at low pore hydration ($\vartheta_{\mathrm{M}} = 0.10$), $g(r)$ shows a peak at $r \cong 0.18$ nm due to the hydrogen bonds between water molecules and inner silanol nests, which induce local water adsorption and thus reduced solvent mobility ($i.e.$, D). The peaks at $r \cong 0.3$ nm, instead, are located in the center of the zeolite's pore, and they indicate the accessible volume of the pore where water-water interactions are predominant. At large pore hydration ($\vartheta_{\mathrm{M}} = 0.95$), similar $g(r)$ for the pristine and defected MFI are observed. At large pore hydration, the peak at $r \cong 0.18$ nm (water-zeolite interactions) is still visible for the defected case; however, the relative magnitude is drastically decreased when compared to the central peak at $r \cong 0.3$ nm (water-water interactions).

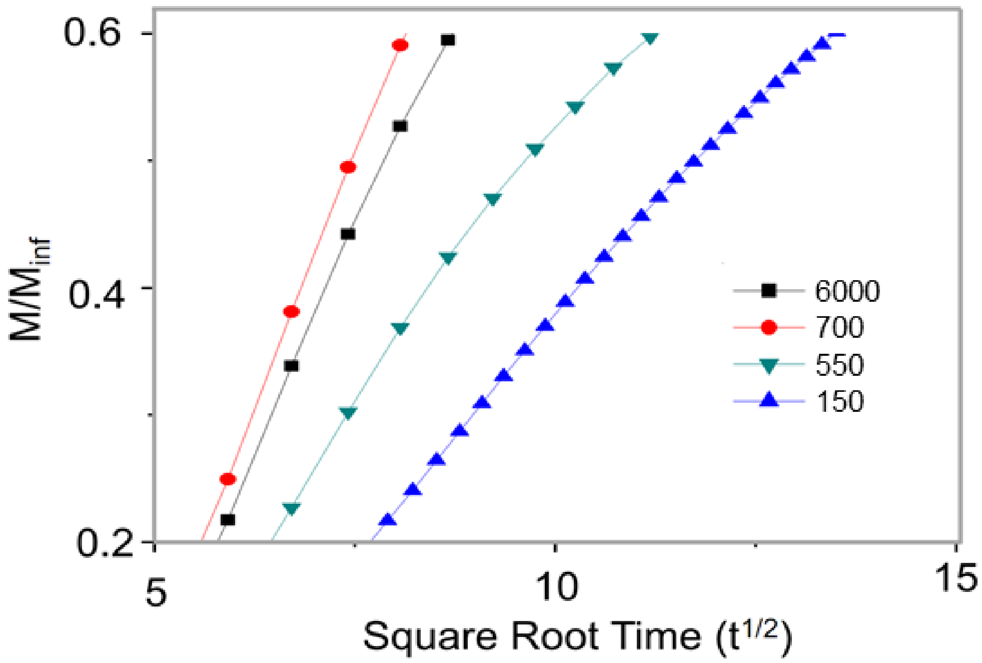

Supplementary Figure 8. Experimental adsorption curves. Example adsorption curves for various sized MFI zeolites taken at a relative pressure of 0.4. The approximate linear response indicates a Fickian diffusion process. The slopes of these curves were used to estimate the effective diffusivity (see equation (1) in reference 3), the average of which is plotted in **Fig. 4** of the manuscript. Symbols (V/S_p) are expressed in nm.

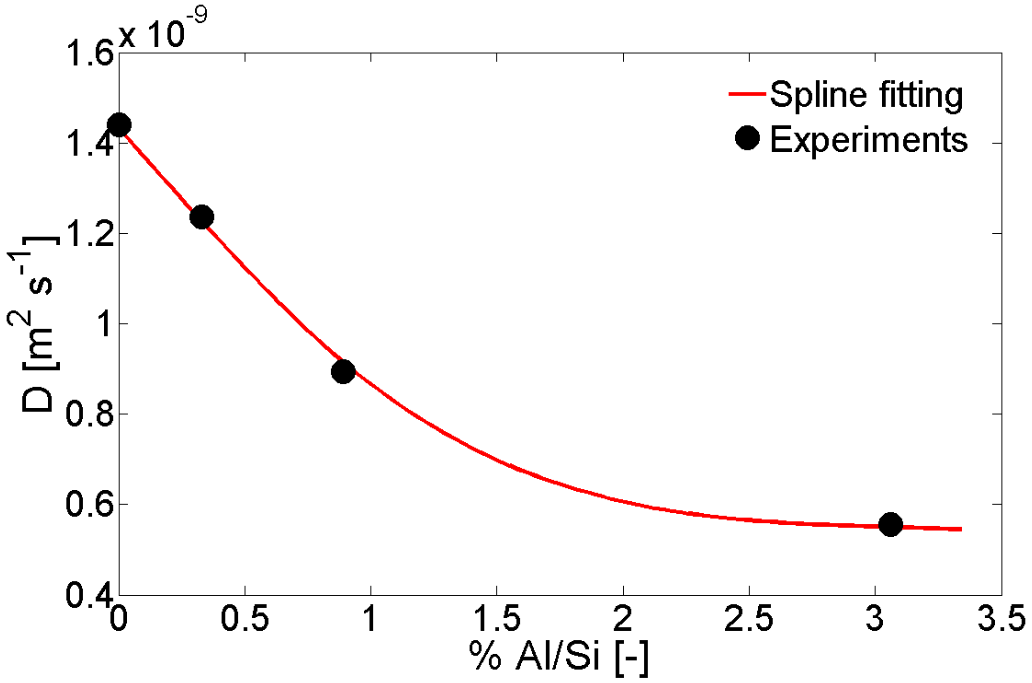

Supplementary Figure 9. Water diffusion and zeolite hydrophilicity. Self-diffusion coefficients for water confined in MFI zeolites with different defect concentrations: MD simulations (black dots) and curve fitting by spline function (red line) are both shown. The reported values are averaged in the $0.4 < p/p_0 < 1$ range and rescaled (*i.e.*, $D_{\mathrm{MD}}: D_{\mathrm{B,MD}} = D_{\mathrm{EXP}}: D_{\mathrm{B,EXP}}$, being $D_{\mathrm{B,MD}} = 3.54 \times 10^{-9}$ m^2 s^{-1} the bulk self-diffusivity for TIP4P water model at 300 K) by the experimental self-diffusivity of bulk water at 300 K (*i.e.*, $D_{\mathrm{B,EXP}} = 2.30 \times 10^{-9}$ m^2 s^{-1}).

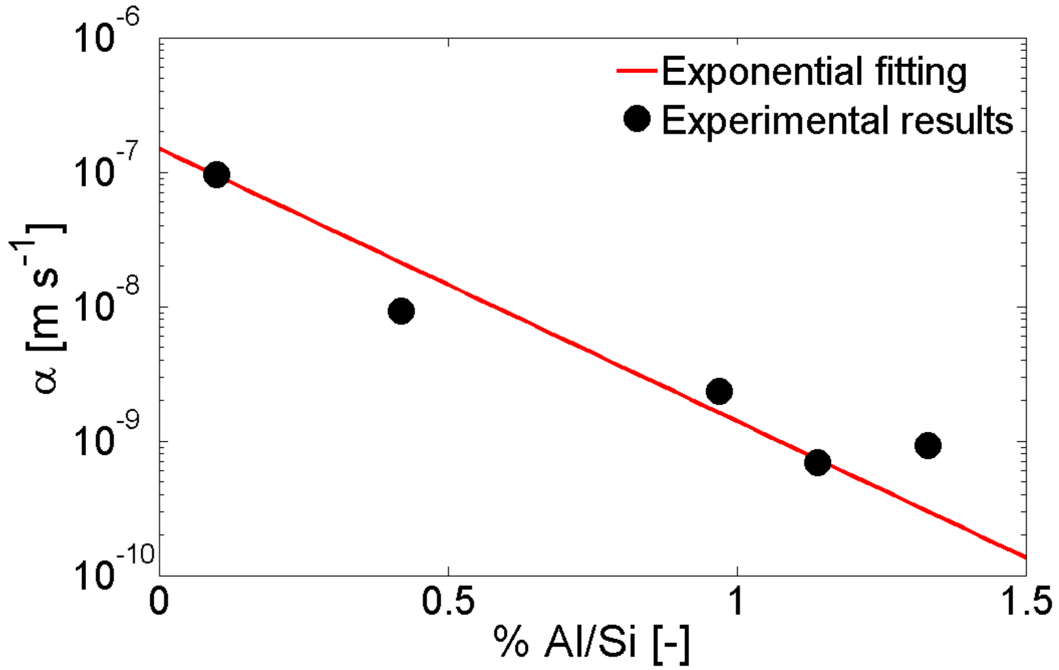

Supplementary Figure 10. Surface permeability and zeolite hydrophilicity. Surface permeability for MFI membranes with increasing amount of hydrophilic defects: experimental results (black dots) are fitted by an exponential curve (red line, see Supplementary Equation (12)). [3] Results are obtained by regression (equation (4) in the main text), where D_0 and V/S_{p} are experimentally measured [3] and D comes from MD simulations (see **Supplementary Fig. 9**).

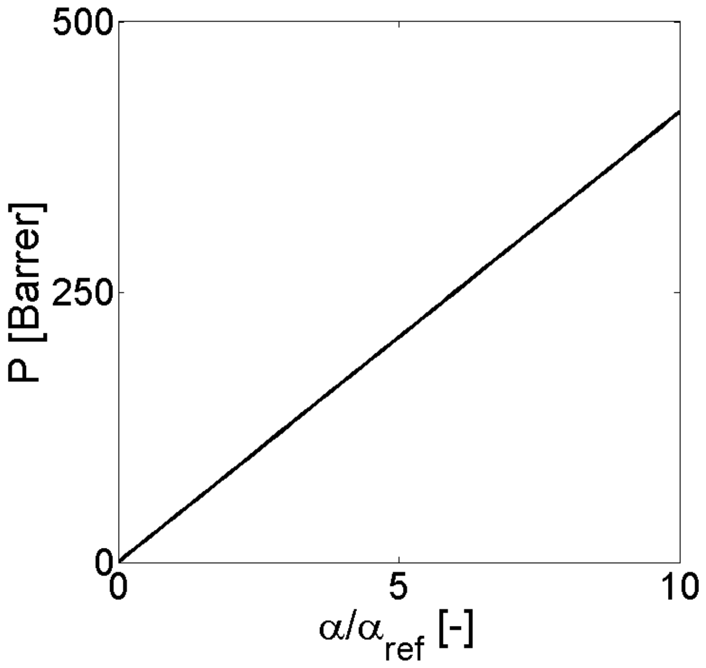

Supplementary Figure 11. Membrane permeability enhancement. Modeling predictions (equation (4) in the main text) for the membrane permeability of silicalite-1 in case of increased surface permeability ($\alpha/\alpha_{\mathrm{ref}}$), where $\alpha_{\mathrm{ref}} = 5.0 \times 10^{-9}$ m s^{-1} is the experimentally measured one.

Supplementary Notes

Supplementary Note 1. Self-diffusion of bulk water

Bulk water is simulated in order to have a reliable term of comparison for the results obtained in the confined setups. A simulation box of $5 \times 5 \times 5$ nm^3 with 2228 TIP4P water molecules is generated, and periodic boundary conditions are imposed along all Cartesian directions. Simulations are then performed in NPT ensemble (velocity rescaling thermostat at $T = 300$ K [8]; isotropic Parrinello-Rahman barostat [9]). First, the self-diffusion coefficient obtained at $p = 1$ MPa is measured as 3.64×10^{-9} m^2 s^{-1}, which is in good agreement with usual values for TIP4P model. [10] Then, a few larger pressures are tested, namely 25, 50, 75, 125 and 200 MPa, in order to measure the self-diffusivity of pressurized water, as in the case of infiltration experiments. Results indicate that D_B is almost constant in the $p = 25 \div 200$ MPa range, namely $D_{\mathrm{B}} = 3.54 \times 10^{-9} \pm 2.5\%$ m^2 s^{-1}, showing a $D_B - p$ trend similar to the experimental one. [11]

Supplementary Note 2. Interaction energies

The water-water and water-zeolite specific interaction energies are defined as:

$$E_{\mathrm{ww}} = \frac{U_{\mathrm{LJ-ww}} + U_{\mathrm{C-ww}}}{\omega N_{\mathrm{UC}}}, \quad (1)$$

and

$$E_{\mathrm{wz}} = \frac{U_{\mathrm{LJ-wz}} + U_{\mathrm{C-wz}}}{\omega N_{\mathrm{UC}}}, \quad (2)$$

respectively. In Supplementary Equations (1) and (2): ω is the amount of infiltrated water molecules per unit cell; N_{UC} is the amount of MFI unit cells in the membrane; $U_{\mathrm{LJ-wz}}$ and $U_{\mathrm{LJ-ww}}$ are the interaction energies between water-zeolite and water-water atoms due to

Lennard-Jones potential (see **Supplementary Fig. 5**), respectively; $U_{\mathrm{C-wz}}$ and $U_{\mathrm{C-ww}}$ are the interaction energies between water-zeolite and water-water atoms due to Coulomb potential (see **Supplementary Fig. 5**), respectively. Note that the calculation of the overall interaction energies U only takes into account infiltrated water molecules.

It is then possible to interpret the coverage dependence of D shown in **Fig. 2b** in terms of a two-phase system. At low concentrations, which correspond to pore coverage of a single surface monolayer or less, water coexists in either an adsorbed (on the pore surface) or a vapour (at the pore axis) phase. Therefore, the molecular diffusion consists of two contributions. [12,13] The first one is Knudsen diffusion, which is a sequence of collisions either with the pore walls or between the water molecules in the vapour phase. The second mode is surface diffusion, which proceeds by a hopping of molecules along the surface. Fast molecular exchange between the two phases ensures that the overall D consists of a parallel process, in which contributions from the two phases simply sum together. [12,13] At larger pore hydrations, instead, capillary-condensation is eventually attained. Therefore, the diffusion contribution of the liquid phase becomes predominant and a two-phase system is no more observed. The liquid diffusion is then only influenced by the tortuosity of the diffusion path, which is related to the configuration of the pores network in the crystal and can be analytically estimated. [12]

Supplementary Note 3. Diffusion resistance model

The basis of equation (4) in the main text is depicted by the schematic in **Fig. 3c**, where water molecules are subject to a series of diffusion resistances while diffusing through the zeolite specimen: (1) starting from bulk conditions, the water molecule (2) "sticks" to the zeolite surface, then (3) enters in an open pore and (4) diffuses through it. Sticking probability

(probability for a fluid molecule to adsorb to a solid surface upon colliding with it) and surface barriers (planes of dramatically reduced permeability, e.g. external pore blockage or narrowing) can be generally considered as complementary phenomena in determining the overall surface diffusion resistance of fluid molecules entering nanopores (diffusion steps 2 and 3 in **Fig. 3c**). However, current experiments and simulations do not allow to clearly distinguish between sticking resistance (which has not a general, analytical formulation) and surface barriers diffusion resistances; therefore, in equation (4) in the main text we chose to approximate the series of diffusion resistances as (i) surface barrier resistance and (ii) intracrystalline (volumetric) resistance.

First, let us recall the definition of surface permeability (α), namely the proportionality factor between fluid flux and difference in concentrations on either sides of a surface barrier: [14,15]

$$j_x = -\alpha(c_\mathrm{l} - c_\mathrm{r}), \tag{3}$$

being j_x the fluid flux through the barrier, c_l and c_r concentrations on the left and right sides of the barrier, respectively. Second, Fick's law relates the diffusive flux of fluid to a concentration gradient at steady state:

$$\mathbf{J} = -D_\mathrm{T}\nabla c, \tag{4}$$

being D_T the Fick's diffusion coefficient and c the fluid concentration. In zeolite pores, fluid transport can be approximated as a one-dimensional diffusion (*e.g.*, along x axis); therefore, Supplementary Equation (4) can be reduced to

$$j_x = -D_\mathrm{T}\frac{\partial c}{\partial x}, \tag{5}$$

where $\frac{\partial c}{\partial x}$ refers to the concentration gradient along the pore length.

Let us now consider the transport of water molecules from bulk conditions (step 1 in **Fig. 3c**, fluid concentration c_1) into zeolite framework (step 4 in **Fig. 3c**, fluid concentration c_3). Hence,

the water flux through the zeolite sample (j_x) during the uptake process can be expressed by Supplementary Equations (3) and (5) as

$$j_x = -\alpha(c_1 - c_2) \tag{6}$$

and

$$j_x = -D_\mathrm{T}\frac{(c_2 - c_3)}{L}, \tag{7}$$

respectively, where $c_1 = c_\mathrm{l}$, $c_2 = c_\mathrm{r}$ and L is the pore length. By imposing the continuity of j_x through the zeolite pores, Supplementary Equations (6) and (7) can be then combined as:

$$j_x = -\left(\frac{1}{\alpha} + \frac{L}{D_\mathrm{T}}\right)^{-1}(c_1 - c_3). \tag{8}$$

Hence, the water transport into zeolite pores is determined by an effective diffusion resistance ($R_\mathrm{eff} = L/D_\mathrm{eff}$), which – analogously to the lumped element models adopted for electric or thermal applications – arises from a series of surface and volume resistances to diffusion, namely

$$R_\mathrm{eff} = \frac{1}{\alpha} + \frac{L}{D_\mathrm{T}}. \tag{9}$$

At low hydration regimes, Barrer approximation holds and thus $D_\mathrm{T} \cong \Gamma D$ (*i.e.*, Darken's equation [14]), where Γ is the thermodynamic factor and D the self-diffusion coefficient. The water transport in uptake experiments can be finally expressed in terms of the effective diffusivity (D_eff), that is

$$D_\mathrm{eff} \cong \left(\frac{1}{\alpha L} + \frac{1}{\Gamma D}\right)^{-1}. \tag{10}$$

Multiplying by Γ both numerator and denominator and recalling that $L = V/S_\mathrm{p}$ in the considered zeolite samples yield finally to the equation (4) reported in main text, namely

$$D_\mathrm{eff} \cong \Gamma\left(\frac{\Gamma}{\alpha V/S_\mathrm{p}} + \frac{1}{D}\right)^{-1}. \tag{11}$$

Supplementary Note 4. Comparison with literature results

The computed α can be compared with previous works in the literature where zeolites (or Metal-Organic Frameworks, MOFs) were typically infiltrated by light hydrocarbons. For example, Chemelik *et al.* reported α in the range 10^{-8} to 10^{-6} m s^{-1} for the uptake of methanol in ferrierite zeolite and 10^{-9} to 10^{-7} m s^{-1} for propane in MOF, according to different relative concentrations; [16] similar results were also found by Heinke *et al.* in case of methanol uptakes into ferrierite. [17] Recently, Saint Remi *et al.* experimentally evaluated α and D for commercial zeolites (SAPO-34) intruded by methanol: while α spreads over almost two orders of magnitude (*i.e.*, from 10^{-9} to 10^{-7} m s^{-1}), D shows substantially unchanged values. [18]

The large variety of reported α values can be mainly attributed to the different synthesis, storage and experimental conditions to which are subjected zeolite/MOF samples. In particular, a progressive degradation of surface permeability with storage time was found in several nanoporous samples. For example, Chemelik *et al.* noticed a 5-fold decrease (from 11.2×10^{-9} m s^{-1} to 2.3×10^{-9} m s^{-1}) in the surface permeability of propane in MOF Zn(tbip) due to an increased storage time of the sample in ambient atmosphere; [19] similarly, Guendré *et al.* reported up to one order of magnitude drop in uptake kinetics with storage time (cyclohexane uptake in silicalite-1). [20] This “ageing” effect of zeolite/MOF surfaces is particularly pronounced in case of expositions to water-containing environments, because of the high reactivity of water molecules with the incomplete terminals of zeolite/MOF surfaces. [19,21,22] These evidences are in line with the relatively low α values reported in this work, where MFI samples were totally immersed in water during uptake experiments.

Despite surface barriers are a peculiar property of each zeolite sample, recent works have demonstrated that surface permeation and intracrystalline diffusion are governed by identical

fundamental transport mechanism. [23] In fact, both surface and volumetric transport phenomena were observed to show the same activation energy, being α/D independent from guest molecules type, loading and equilibrium/non-equilibrium conditions. [24,25] These observations actually led to the current interpretation of surface barriers as pore blockage or narrowing phenomena. Furthermore, α has revealed dependence with environment pressure and post-synthesis treatments (e.g., etching), which may both alter the amount of pore mouth openings. [18,26]

Supplementary Note 5. Pore blockage/narrowing modeling

In contrast with the analysis of water diffusion within the regular network of zeolite's nanopores, ideal surface structures cannot be defined *a priori* without some arbitrariness. Current experimental techniques are unable to fully characterize the exact nature of surface terminations responsible of surface diffusion resistances, therefore limiting the possibility to compute α by mechanistic considerations. Furthermore, surface barriers can depend strongly on the conditions under which zeolites have been synthesized, stored, prepared for measurement and even on the permeation measurements themselves. [27,28] To interpret the experimental/numerical values of α, some authors have suggested empirical models based on the probability of both channel mouth opening and intracrystalline channel connection. [27] These models are typically tailored for particular sets of experiments, and require parameters to be empirically fitted; [25,29,30] hence, a rigorous derivation of predictive models for surface permeability remains an open issue. [18,27]

However, by considering the water uptake experiments in MFI zeolites by Humplik and colleagues (D_{eff}, Γ and V/S_{p} [3]) and the current molecular dynamics results (D), Supplementary Equation (11) allows to find a relation between surface permeability and defects concentration

($\%Al/Si$). Results (see **Supplementary Fig. 10**) show that α undergoes an exponential decrease by increasing the concentration of hydrophilic defects in MFI zeolites, namely

$$\alpha = k_1 \exp(k_2 \times \%Al/Si), \qquad (12)$$

where $k_1 = 1.50 \times 10^{-7}\ \mathrm{m\ s^{-1}}$ and $k_2 = -4.67$ (R^2 = 0.97). In fact, the progressive introduction of hydrophilic defects in MFI zeolites may decrease the fraction of surface pore openings, thus reducing α.

Therefore, the observed D_{eff} reduction in water uptake experiments with defective MFI zeolites should be mainly attributed to surface effects rather to volumetric ones. In fact, defect concentrations ranging from 0.1 to 1.3 $\%Al/Si$ seem to cause a 100-fold α drop (see **Supplementary Fig. 10**, as from experiments), while only a 2-fold D decrease (see **Supplementary Fig. 9**, as from molecular dynamics simulations).

Supplementary Note 6. Perspectives

The reported results unveil a promising future for zeolite-based membranes (see **Supplementary Fig. 11**), because – by reducing surface diffusion resistances – they could potentially achieve orders of magnitude enhancements in membrane permeability respect to state-of-the-art polymeric membranes. To this purpose, in this work we highlight the key role of first understanding the chemical origin of surface barriers (whether they are an intrinsic property of synthesis or are caused by avoidable surface contamination), and then to introduce novel experimental techniques to reducing the hindered transport that occurs at the surface.

On the one hand, molecular-based simulations using zeolites as the membrane material typically utilize a 'perfect' (surface barrier free) crystal as the active membrane. [31,32] Since these simulations have greatly exaggerated the performance of zeolites in comparison to the

experiments, we believe that future simulations should incorporate this non-ideal structure within their model. However, a clear picture of surface structure of zeolites remains a challenge, because current imaging techniques are not yet precise enough to visualize experimentally such subnanometer surface terminations. [23,30] Hence, computational quantum mechanics may be a helpful tool to determine the chemical characteristics of pore blockage or narrowing at the membrane surface, and it could be eventually coupled to molecular dynamics techniques to achieve a more accurate, multiscale simulation of surface diffusion resistances.

On the other hand, this work highlights the need for new surface treatments for zeolite-based membranes, which may reduce the surface pore blockage/narrowing thus enhancing their permeability regardless the presence of intracrystalline defects. For example, faster molecular transport could be attained by post-synthetic processing, such as *ad hoc* surface etching; [23] whereas clean synthesis and storage are also important to avoid the formation of surface barriers during samples' handling and operation. [21]

Supplementary Methods

Molecular dynamics geometry

MFI belongs to a class of zeolites similar to both large-pore Faujasite and small-pore LTA, with pore diameters characterized by intermediate dimensions. [33] MFI zeolite has a orthorhombic crystal structure, with Pnma space group and lattice constants $a = 20.022$, $b = 19.899$ and $c = 13.383$ Å, [34] respectively. The MFI framework contains a network of 3-dimensional channels, which are formed by two intersecting subsystems: straight channels parallel to [010] direction and zig-zag channels parallel to [001], with average pore diameters of about 5.5 Å. The latter configuration implies a 45% porosity of the structure, where inner cavities with 6.36 Å diameter are present at the channel intersections. Note that the characteristic size of the pores allows the diffusion of molecules with diameters less than 4.46 Å, therefore permitting the flow of water while blocking hydrated ions. [35]

Concerning the arrangement of defects in the zeolite structure, the positions of defects are first randomly defined while excluding the most external volumes of the membrane, in order to avoid issues at the periodic boundaries or modify the structure of the surface pore openings. For lab-synthesized MFI zeolites, the presence of hydrophilic point defects can be regulated by the introduction of aluminium atoms promoting an unbound oxygen, which in turn forms a silanol terminal in the structure. [36,37] Taking inspiration from the “silanol nest model” proposed by Caillez and colleagues to simulate general hydrophilic point defects in zeolites, [2,38] in this work the increase in pore hydrophilicity due to Al insertion is mimicked by the introduction of silanols in the MFI structure. In detail, a hydrophilic defect is generated by removing a Si atom of the zeolite framework; the resulting four dangling oxygen atoms are then functionalized as silanols. Clearly, silanol nests and Al defects are different from both structural and chemical point of

view. However, the intracrystalline water transport is mainly dictated by the non-bonded interactions between solid and liquid phase. [39] Moreover, it has been demonstrated that, instead of using different force field parameters for silanol nests (q1) and Al (q2) defects, it is possible to work with a single tunable parameter (Si-OH dipole moment, in this case) to be defined by global comparison with experimental data. [2,38] In fact, the resulting effect (*i.e.*, zeolite hydrophilicity) to be compared with experiments would be q = w1 x q1 + w2 x q2, namely an infinite combination of w1 and w2 weights. This is a simplified *top-down* approach to tune (and validate) molecular dynamics force fields to experimental data, namely an alternative to the *bottom-up* derivation from quantum mechanics simulations.

Molecular dynamics force field

In the zeolite structure, bonded interactions are modelled by two harmonic terms, which are adopted to describe the silicon-oxygen and oxygen-hydrogen interactions. In particular, a bond stretching potential between two bonded atoms i and j at a distance r_{ij} (around the equilibrium distance r_{ij}^0) and a bending angle potential between the two pairs of bonded atoms (i,j) and (j,k) (around the equilibrium angle β_{ijk}^0) are considered as follows:

$$U_{BOND}\left(r_{ij},\vartheta_{ijk}\right)=\frac{1}{2}k_{ij}^{b}(r_{ij}-r_{ij}^{0})^2+\frac{1}{2}k_{ijk}^{\vartheta}(\beta_{ijk}-\beta_{ijk}^{0})^2, \quad (13)$$

where k_{ij}^b and β_{ijk} are taken from reference 40, whereas r_{ij}^0 and β_{ijk}^0 come from the equilibrium structure of MFI zeolite**.** [34] For stability purposes, the atoms of the zeolite framework are restrained to the crystallographic positions ($\mathbf{R}_i$) by means of a harmonic potential:

$$V_{pr}(\mathbf{r}_i)=\frac{1}{2}k_{\mathrm{pr}}|\mathbf{r}_i-\mathbf{R}_i|^2, \quad (14)$$

where $k_{\mathrm{pr}}=5000$ kJ mol^{-1} nm^{-2}. [2,41,42]

Non-bonded interactions among zeolite atoms and between zeolite and water atoms are taken into account by: (i) a Lennard-Jones term (van der Waals interactions)

$$U_{LJ}(r_{ij}) = 4\varepsilon_{ij}\left[\left(\frac{\sigma_{ij}}{r_{ij}}\right)^{12} - \left(\frac{\sigma_{ij}}{r_{ij}}\right)^{6}\right], \quad (15)$$

with ε_{ij} (potential well) and σ_{ij} (van der Waals radius) mixed parameters obtained from Lorentz-Berthelot combination rules

$$\begin{cases} \sigma_{ij} = \frac{1}{2}(\sigma_{ii} + \sigma_{jj}) \\ \varepsilon_{ij} = (\varepsilon_{ii} + \varepsilon_{jj})^{1/2} \end{cases}; \quad (16)$$

(ii) a Coulomb term (electrostatic interactions)

$$U_{\mathrm{C}}(r_{ij}) = \frac{1}{4\pi\varepsilon_0}\frac{q_i q_j}{r_{ij}}, \quad (17)$$

being ε_0 the permittivity in a vacuum, q_i, q_j the partial charge of atoms i and j, respectively. Partial charges in the silanol groups are assigned following the criterion of the overall neutral charge for the entire system, namely $q_{\mathrm{O}} = -q_{\mathrm{Si}}/2$ and $q_{\mathrm{H}} = q_{\mathrm{Si}}/4$.

Initially, both Lennard-Jones and partial charges of MFI zeolite were taken from the values reported by Cailliez *et al.*, [2] where partial charges of silicon, oxygen and hydrogen atoms in the MFI structure are $q_{\mathrm{Si}} = 1.4$ e, $q_{\mathrm{O}} = -0.7$ e and $q_{\mathrm{H}} = 0.35$ e, respectively. The value of q_{Si} was successively tuned to better mimic the adsorption/infiltration isotherms of the zeolites experimentally tested. *Ad hoc* infiltration MD runs (**Supplementary Figs. 1** and **2**) showed that $q_{\mathrm{Si}} = 1.8$ e better reproduced both the experimental infiltration pressure ($\cong 95$ MPa) and the adsorption capacity ($\cong 5$ N/UC, water molecules per unit cell at $p \cong p_0$) of water in the considered silicalite-1 samples. Further details on these infiltration MD runs are reported in reference 7 and in the section below. The TIP4P water model was used for the solvent, [43] since it

is well-reported that it reproduces the transport properties of water molecules confined structures. [2] The intramolecular interactions of water molecules were fixed by LINCS algorithm, [44] to increase the simulation time step.

Lennard-Jones potentials are treated with a twin-range cut-off modified by a shift function (1.0 nm cut-off distance), whereas the Particle-Mesh Ewald (PME) algorithm with 1 nm real-space cut-off and 0.12 nm reciprocal space gridding is chosen for electrostatic interactions. [45] Long-range dispersion corrections are applied to avoid energy artefacts.

Water infiltration in silicalite-1 by MD

A membrane made out of $2 \times 3 \times 3$ orthorhombic unit cells of MFI zeolite (*i.e.*, $4 \times 6 \times 4\ \mathrm{nm}^3$ dimensions along the x, y, z directions, see **Supplementary Fig. 1**) is considered for the MD infiltration experiments needed for tuning the Coulomb interactions. [7,34] The pristine MFI zeolite (silicalite-1) is made out of only silicon and oxygen atoms, thus showing hydrophobic behaviour. [4] Once the pristine MFI geometry is generated, the membrane is placed in the centre of a $4 \times 6 \times 34\ \mathrm{nm}^3$ simulation box, so that the water-zeolite interface, which is functionalized by silanol terminals, is normal to z axis. The resulting simulation box is then solvated by TIP4P water molecules, and $\cong 30000$ molecules are typically added. Periodic boundary conditions are considered along x, y, z directions. After the energy minimization of the geometry, velocities are initialized by a Maxwell distribution (300 K). In order to avoid variations of the membrane position, harmonic position restraints (spring constant 5000 kJ mol^{-1} nm^{-2}) are then applied to the atoms of the membrane. The system temperature is then equilibrated at 300 K and the water pressure stabilized around 0.1 MPa by successive NVT and NPT runs, where a Berendsen thermostat and barostat are used. [46] The equilibration of the structure is typically carried out for

1.5 ns. Finally, infiltration runs are performed in NPT ensemble (velocity rescaling thermostat with $T = 300$ K and time constant $\tau = 0.1$ ps; [8] isotropic Parrinello-Rahman barostat with p water pressure to be tested and time constant $\tau = 2$ ps [9]), so that water molecules can intrude in the initially empty zeolite membrane until equilibrium conditions are reached, typically after 10-35 ns. MD simulations are carried out by GROMACS (leap-from algorithm; time step 2 fs). [47]

Supplementary References

1 Pettersen, E. F. *et al.* UCSF Chimera-A visualization system for exploratory research and analysis. *J. Comput. Chem.* **25**, 1605-1612 (2004).

2 Cailliez, F., Stirnemann, G., Boutin, A., Demachy, I. & Fuchs, A. H. Does water condense in hydrophobic cavities? A molecular simulation study of hydration in heterogeneous nanopores. *J. Phys. Chem. C* **112**, 10435-10445 (2008).

3 Humplik, T., Raj, R., Maroo, S. C., Laoui, T. & Wang, E. N. Effect of Hydrophilic Defects on Water Transport in MFI Zeolites. *Langmuir* **30**, 6446-6453 (2014).

4 Humplik, T., Raj, R., Maroo, S. C., Laoui, T. & Wang, E. N. Framework water capacity and infiltration pressure of MFI zeolites. *Microporous Mesoporous Mater.* **190**, 84-91 (2014).

5 Olson, D., Haag, W. & Borghard, W. Use of water as a probe of zeolitic properties: interaction of water with HZSM-5. *Microporous Mesoporous Mater.* **35**, 435-446 (2000).

6 Ramachandran, C. E., Chempath, S., Broadbelt, L. J. & Snurr, R. Q. Water adsorption in hydrophobic nanopores: Monte Carlo simulations of water in silicalite. *Microporous Mesoporous Mater.* **90**, 293-298 (2006).

7 Fasano, M., Borri, D., Chiavazzo, E. & Asinari, P. Protocols for atomistic modeling of water uptake into zeolite crystals for thermal storage and other applications. *Appl. Therm. Eng.* **101**, 762-769 (2016).

8 Bussi, G., Donadio, D. & Parrinello, M. Canonical sampling through velocity rescaling. *J. Chem. Phys.* **126**, 014101 (2007).

9 Parrinello, M. & Rahman, A. Polymorphic transitions in single crystals: A new molecular dynamics method. *J. Appl. Phys.* **52**, 7182-7190 (1981).

10 van der Spoel, D., van Maaren, P. J. & Berendsen, H. J. A systematic study of water models for molecular simulation: derivation of water models optimized for use with a reaction field. *J. Chem. Phys.* **108**, 10220-10230 (1998).

11 Krynicki, K., Green, C. D. & Sawyer, D. W. Pressure and temperature dependence of self-diffusion in water. *Faraday Discuss. Chem. Soc.* **66**, 199-208 (1978).

12 D'Orazio, F., Bhattacharja, S., Halperin, W. P. & Gerhardt, R. Enhanced self-diffusion of water in restricted geometry. *Phys. Rev. Lett.* **63**, 43 (1989).

13 Valiullin, R., Kortunov, P., Kärger, J. & Timoshenko, V. Concentration-dependent self-diffusion of liquids in nanopores: A nuclear magnetic resonance study. *J. Chem. Phys.* **120**, 11804-11814 (2004).

14 Kärger, J. *et al.* Microimaging of transient guest profiles to monitor mass transfer in nanoporous materials. *Nat. Mater.* **13**, 333-343 (2014).

15 Kärger, J., Bülow, M., Millward, G. & Thomas, J. A phenomenological study of surface barriers in zeolites. *Zeolites* **6**, 146-150 (1986).

16 Chmelik, C. *et al.* Ensemble measurement of diffusion: novel beauty and evidence. *ChemPhysChem* **10**, 2623-2627 (2009).

17 Heinke, L., Kortunov, P., Tzoulaki, D. & Kärger, J. Exchange dynamics at the interface of nanoporous materials with their surroundings. *Phys. Rev. Lett.* **99**, 228301 (2007).

18 Saint Remi, J. C. *et al.* The role of crystal diversity in understanding mass transfer in nanoporous materials. *Nat. Mater.* **15**, 401-406 (2016).

19 Chmelik, C. *et al.* Mass transfer in a nanoscale material enhanced by an opposing flux. *Phys. Rev. Lett.* **104**, 085902 (2010).

20 Gueudré, L., Bats, N. & Jolimaître, E. Effect of surface resistance on cyclohexane uptake curves in Silicalite-1 crystals. *Microporous Mesoporous Mater.* **147**, 310-317 (2012).

21 Heinke, L., Gu, Z. & Wöll, C. The surface barrier phenomenon at the loading of metal-organic frameworks. *Nat. Commun.* **5**, 4562 (2014).

22 Tzoulaki, D., Schmidt, W., Wilczok, U. & Kärger, J. Formation of surface barriers on silicalite-1 crystal fragments by residual water vapour as probed with isobutane by interference microscopy. *Microporous Mesoporous Mater.* **110**, 72-76 (2008).

23 Teixeira, A. R. *et al.* 2D Surface Structures in Small Zeolite MFI Crystals. *Chem. Mater.* **27**, 4650-4660 (2015).

24 Hibbe, F. *et al.* The nature of surface barriers on nanoporous solids explored by microimaging of transient guest distributions. *J. Am. Chem. Soc.* **133**, 2804-2807 (2011).

25 Teixeira, A. R. *et al.* Dominance of Surface Barriers in Molecular Transport through Silicalite-1. *J. Phys. Chem. C* **117**, 25545-25555 (2013).

26 Chang, C.-C., Teixeira, A. R., Li, C., Dauenhauer, P. J. & Fan, W. Enhanced Molecular Transport in Hierarchical Silicalite-1. *Langmuir* **29**, 13943-13950 (2013).

27 Heinke, L. & Kärger, J. Correlating surface permeability with intracrystalline diffusivity in nanoporous solids. *Phys. Rev. Lett.* **106**, 074501 (2011).

28 Kärger, J. In-depth study of surface resistances in nanoporous materials by microscopic diffusion measurement. *Microporous Mesoporous Mater.* **189**, 126-135 (2013).

29 Schüring, A., Gulín-González, J., Vasenkov, S. & Fritzsche, S. Quantification of the mass-transfer coefficient of the external surface of zeolite crystals by molecular dynamics simulations and analytical treatment. *Microporous Mesoporous Mater.* **125**, 107-111 (2009).

30 Teixeira, A. R. *et al.* On Asymmetric Surface Barriers in MFI Zeolites Revealed by Frequency Response. *J. Phys. Chem. C* **118**, 22166-22180 (2014).

31 Turgman-Cohen, S., Araque, J. C., Hoek, E. M. & Escobedo, F. A. Molecular dynamics of equilibrium and pressure-driven transport properties of water through LTA-type zeolites. *Langmuir* **29**, 12389-12399 (2013).

32 Liu, Y. & Chen, X. High permeability and salt rejection reverse osmosis by a zeolite nano-membrane. *Phys. Chem. Chem. Phys.* **15**, 6817-6824 (2013).

33 Kokotailo, G., Lawton, S. & Olson, D. Structure of synthetic zeolite ZSM-5. *Nature* **272**, 437-438 (1978).

34 Van Koningsveld, H., Jansen, J. & Van Bekkum, H. The monoclinic framework structure of zeolite H-ZSM-5. Comparison with the orthorhombic framework of as-synthesized ZSM-5. *Zeolites* **10**, 235-242 (1990).

35 Humplik, T. *et al.* Nanostructured materials for water desalination. *Nanotechnology* **22**, 292001 (2011).

36 Chang, C. D., Chu, C. T., Miale, J. N., Bridger, R. F. & Calvert, R. B. Aluminum insertion into high silica zeolite frameworks. 1. Reaction with aluminum halides. *J. Am. Chem. Soc.* **106**, 8143-8146 (1984).

37 Redondo, A. & Hay, P. J. Quantum chemical studies of acid sites in zeolite ZSM-5. *J. Phys. Chem.* **97**, 11754-11761 (1993).

38 Trzpit, M. *et al.* The Effect of Local Defects on Water Adsorption in Silicalite-1 Zeolite: A Joint Experimental and Molecular Simulation Study. *Langmuir* **23**, 10131-10139 (2007).

39 Chiavazzo, E., Fasano, M., Asinari, P. & Decuzzi, P. Scaling behaviour for the water transport in nanoconfined geometries. *Nat. Commun.* **5**, 4495 (2014).

40 Lopes, P. E., Murashov, V., Tazi, M., Demchuk, E. & MacKerell, A. D. Development of an empirical force field for silica. Application to the quartz-water interface. *J. Phys. Chem. B* **110**, 2782-2792 (2006).

41 Desbiens, N., Boutin, A. & Demachy, I. Water condensation in hydrophobic silicalite-1 zeolite: a molecular simulation study. *J. Phys. Chem. B* **109**, 24071-24076 (2005).

42 Yanai, N. *et al.* Gas detection by structural variations of fluorescent guest molecules in a flexible porous coordination polymer. *Nat. Mater.* **10**, 787-793 (2011).

43 Jorgensen, W. L., Chandrasekhar, J., Madura, J. D., Impey, R. W. & Klein, M. L. Comparison of simple potential functions for simulating liquid water. *J. Chem. Phys.* **79**, 926-935 (1983).

44 Hess, B., Bekker, H., Berendsen, H. J. & Fraaije, J. G. LINCS: a linear constraint solver for molecular simulations. *J. Comput. Chem.* **18**, 1463-1472 (1997).

45 Allen, M. P. & Tildesley, D. J. *Computer simulation of liquids*. (Oxford university press, 1989).

46 Berendsen, H. J., Postma, J. P. M., van Gunsteren, W. F., DiNola, A. & Haak, J. Molecular dynamics with coupling to an external bath. *J. Chem. Phys.* **81**, 3684-3690 (1984).

47 Hess, B., Kutzner, C., Van Der Spoel, D. & Lindahl, E. GROMACS 4: Algorithms for highly efficient, load-balanced, and scalable molecular simulation. *J. Chem. Theory Comput.* **4**, 435-447 (2008).
